# Comparing acute effects of heavy resistance, plyometric, and complex training on post-activation performance enhancement in elite swimmers: a randomized controlled trial

**DOI:** 10.3389/fphys.2026.1748244

**Published:** 2026-03-16

**Authors:** Zhili Ma, Chuanbo Zheng, Tao Gao, Ziren Zhao, Xin Zheng, Ting Liao, Yong “Tai” Wang

**Affiliations:** 1 College of Sports and Health, Chengdu University of Traditional Chinese Medicine, Chengdu, Sichuan, China; 2 Sport Training School, Wuhan Sports University, Wuhan, China; 3 College of Physical Education and Health Science, Chongqing Normal University, Chongqing, China; 4 College of Health Sciences and Technology, Rochester Institute of Technology, Rochester, NY, United States

**Keywords:** complex training, elite swimmers, heavy resistance training, plyometric training, post-activation performance enhancement

## Abstract

**Background:**

Post-activation performance enhancement (PAPE) is an emerging strategy for optimizing pre-competition warm-up in elite swimming. However, substantial heterogeneity exists across studies due to inconsistent load standardization methodologies.

**Objective:**

This randomized controlled trial aimed to examine the acute effects of three PAPE training modalities, heavy resistance training (HRT), plyometric training (PLY), and complex training (COM), on swim start performance and lower body power in elite swimmers, utilizing session rating of perceived exertion (sRPE) for load equalization.

**Methods:**

Forty-seven first-class swimmers (mean age 21.21 ± 0.69 years; training experience 8.08 ± 0.91 years) were randomly allocated to control (n = 11), heavy resistance training (n = 11), plyometric training (n = 12), or complex training (n = 13) groups. All interventions were standardized to achieve equivalent session rating of perceived exertion-time load (sRPE-TL) of 70–80 arbitrary units. The primary outcome was 15-m swim start time (T15m). Secondary outcomes included force platform variables (peak horizontal force, average propulsive force, propulsive impulse, take-off velocity) and land-based power measures (countermovement jump height and peak power). Performance assessments were conducted at 3, 6, 9, and 12 min post-intervention across four separate testing sessions. Effect sizes were calculated using Cohen’s d for within-group pre-post comparisons.

**Results:**

Mixed-model ANOVA revealed significant group × time interactions for T15m (F (3,43) = 2.339, P = 0.024, η^2^p = 0.14), peak horizontal force (F (3,43) = 19.407, P < 0.001, η^2^p = 0.58), average propulsive force (F (3,43) = 7.005, P < 0.001, η^2^p = 0.33), propulsive impulse (F (3,43) = 21.777, P < 0.001, η^2^p = 0.60), take-off velocity (F (3,43) = 23.148, P < 0.001, η^2^p = 0.62), CMJ height (F (3,43) = 2.884, P = 0.032, η^2^p = 0.17), and peak power (F (3,43) = 10.188, P < 0.001, η^2^p = 0.42). COM induced the largest improvements compared to CON, with T15m decresing by 3.00% (ES = 1.79, P < 0.001), peak horizontal force increasing by 5.14% (ES = 4.95, P < 0.001), average propulsive force by 8.48% (ES = 1.71, P < 0.001), propulsive impulse by 8.57% (ES = 3.46, P < 0.001), and take-off velocity by 6.41% (ES = 3.06, P < 0.001). Distinct temporal profiles emerged: PLY peaked at 6 min, HRT sustained effects through 12 min, while COM demonstrated optimal windows at 9–12 min sRPE-TL standardization successfully eliminated between-group load variability (CV < 8%, F (2,33) = 1.23, P = 0.297).

**Conclusion:**

Under sRPE-TL-standardized conditions, complex training elicited greater PAPE responses, with distinct optimal time windows among modalities (PLY: 6 min; HRT: 12 min; COM: 9–12 min), supporting individualized pre-race warm-up programming.

## 1 Introduction

In swimming competition, the 15-m start phase accounting for 26%–30% of total 50-m freestyle time (Garcia-Ramos et al., 2016). At elite competitions such as the Olympic Games, gold medalists typically finish only 0.72 s ahead of eighth-place finishers (approximately 2.92% of winning time) (Đurović et al., 2022). Consequently, maximizing neuromuscular activation during pre-race warm-up to achieve optimal explosive capacity at the starting signal is now a major focus in sport science. Post-activation performance enhancement (PAPE), as an innovative strategy transcending traditional warm-up paradigms, is influencing the theoretical framework and practical applications of pre-competition preparation in swimming (Blazevich and Babault, 2019; Cuenca-Fernández et al., 2022).

PAPE refers to a physiological phenomenon whereby subsequent explosive performance is significantly enhanced following high-intensity muscle contraction activity after an appropriate recovery interval (Tillin and Bishop, 2009; Sale, 2002). Unlike the traditional concept of post-activation potentiation (PAP), which focuses primarily on muscle contractile mechanics, PAPE emphasizes improvements in actual motor performance, thereby better reflecting elite performance demands (Blazevich and Babault, 2019). The core mechanisms involve enhanced motor neuron excitability and recruitment (Aagaard et al., 2002; Del Vecchio et al., 2019) alongside myosin light chain phosphorylation that increases calcium sensitivity by 20%–30% (Sweeney et al., 1993; Grange et al., 1993). Recent work has further elucidated these mechanisms, demonstrating that PAPE effects extend to both explosive performance and neuromuscular control, including improvements in postural stability and jumping performance (Karabel and Makaracı, 2025). Over the past 5 years, PAPE research in land-based sports such as sprinting, jumping, and throwing has increased considerably. While land-based sports show robust effects (d = 0.77–1.2) (Terbalyan et al., 2025; Gautam et al., 2024), swimming applications remain limited given the sport’s unique aquatic demands (Seitz and Haff, 2016; Cuenca-Fernández et al., 2017).

Currently, training modalities used to induce PAPE effects primarily include heavy resistance training (HRT), plyometric training (PLY), and complex training (COM). Heavy resistance training activates high-threshold motor units through high-load resistance movements typically ≥85%, one-repetition maximum (1RM), inducing sustained neural drive enhancement with effects persisting for 8–12 min (16, 17. In 2022, Đurović et al. demonstrated in national-level swimmers that 4–8 min following three sets × 3 repetitions × 87% 1RM back squats, 15-m start time improved by 1.7%–2.0% (effect size d = 0.65–0.82) (Đurović et al., 2022). Plyometric training, utilizing rapid explosive movements of the stretch-shortening cycle (SSC), preferentially recruits fast-twitch muscle fibers and potentiates stretch reflex sensitivity, achieving peak effects at 3–6 min post-intervention (Kilduff et al., 2008; Gołaś et al., 2016). Zhou’s recent investigation demonstrated that merely three sets × 5 repetitions of drop jumps significantly enhanced swim start performance within 6 min (15-m time reduced by 1.9%, ES = −0.47), and due to its simplicity and time efficiency, this study has been identified as an promising approach for pre-race warm-up (Zhou et al., 2024). Complex training combines characteristics of both modalities, theoretically achieving synergistic enhancement of “sustained activation + explosive amplification” through sequential integration of heavy resistance and ballistic movements (McBride et al., 2005; Bevan et al., 2010). However, whether this theoretical advantage translates into actual performance improvements in swimming-specific contexts, as well as comparative aspects including optimal time windows, effect sustainability, and individual response variability among the three training modalities, remain notable gaps requiring investigation.

Critically, previous PAPE research has three notable limitations that compromise the reliability, comparability, and translational value of findings. First, the lack of load standardization represents an important contributing factor to methodological limitations. Most studies design training protocols based solely on objective load parameters (e.g., weight percentages, jump repetitions) (Kilduff et al., 2007; Koźlenia and Domaradzki, 2023), neglecting vast inter-individual differences in training tolerance, the same 85% 1RM load may produce diametrically opposite effects ranging from “appropriate activation” to “excessive fatigue” in different athletes (Wilson et al., 2013). Foster in their comprehensive review on session rating of perceived exertion (sRPE) application, explicitly stated that sRPE integrates multidimensional training stress including metabolic, neural, and psychological factors, demonstrating correlation coefficients of 0.70–0.90 with objective physiological indicators (heart rate, blood lactate), establishing it as the widely accepted method for training load monitoring (Foster et al., 2021; Haddad et al., 2017). Coyne further emphasized that sRPE, compared to objective indicators, better reflects “allostatic load,” particularly demonstrating unique advantages in mixed training incorporating technical, tactical, and strength elements (Coyne et al., 2018). However, to date, no swimming PAPE study has employed sRPE for load standardization, which may constitute the an important contributing factor of substantial heterogeneity (I^2^ = 78–85%) across studies (Terbalyan et al., 2025; Li et al., 2024).

In addition to load standardization challenges, incomplete time window exploration represents another critical methodological limitation in current PAPE research. Existing swimming-specific studies predominantly employ single or dual time-point measurements (Đurović et al., 2022; Seitz and Haff, 2016; Cuenca-Fernández et al., 2017), failing to comprehensively reveal the dynamic temporal progression characteristics of PAPE effects. Cuenca-Fernández in their systematic review on temperature maintenance and PAPE, indicated that optimal effect windows for different training modalities exhibit significant variations (3–20 min) and are modulated by individual factors including strength level, fatigue state, and maturation status (Cuenca-Fernández et al., 2024; Silva et al., 2018). The absence of multi-time-point measurements prevents coaches from precisely determining optimal activation timing, reducing PAPE strategy operability in authentic competition scenarios.

Additionally, superficial mechanism exploration hinders theoretical advancement. Most studies evaluate only endpoint performance indicators (e.g., 15-m time, jump height), lacking systematic measurement of intermediate mechanical parameters (e.g., force plate thrust, Propulsive impulse, take-off velocity) and neuromuscular activity (e.g., electromyography, rate of force development) (Bishop and Middleton, 2013; Radnor et al., 2018), making it difficult to elucidate the complete transmission pathway from neural activation to performance enhancement in PAPE effects.

Therefore, the present study employed controlled load standardization design, utilizing sRPE-time load product (sRPE-TL) as the control indicator, achieving equivalent subjective load intensity across three training modalities through individualized adjustment (target range: 70–80 arbitrary units). Concurrently, a multi-time-point measurement strategy (3, 6, 9, 12 min post-intervention) was performed to provide precise evidence for determining optimal activation time windows. Regarding indicator selection, this study measured not only swimming-specific 15-m start time but also synchronously collected force plate data (peak horizontal force, Average propulsive force, Propulsive impulse, take-off velocity) and land-based explosive power indices (vertical jump height, peak power), constructing a comprehensive evaluation system from neuromuscular activation to actual motor performance, thereby deepening understanding of PAPE effect transfer mechanisms.

Based on these considerations, we tested the following hypotheses: Hypothesis 1: Under load-equivalent conditions (sRPE-TL: 70-80 AU), complex training would induce significantly greater PAPE effects across multiple performance domains compared to single-modality protocols (HRT or PLY); and Hypothesis 2: The three training modalities would exhibit distinct optimal time windows for peak performance enhancement.

## 2 Materials and methods

### 2.1 Study design

**FIGURE 1:**
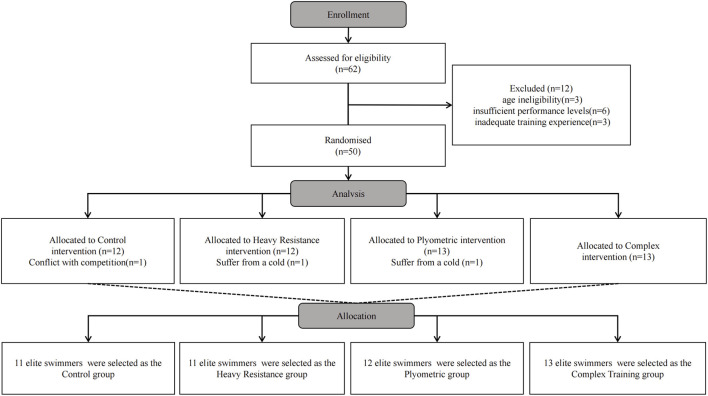
Flow-chart of the randomized trial.

This study employed a single-blind, randomized controlled trial design. Participants meeting inclusion criteria were randomly allocated to CON, HRT, PLY, or COM groups using simple randomization via SPSS-generated random numbers (Figure 1). The investigation was conducted at Wuhan Sports University from September to November 2025. Personnel involved in participant recruitment were not included in randomization table generation. An independent researcher conducted statistical analysis, remaining blinded to intervention allocation. The study received institutional ethics committee approval (approval number: 20250913) and strictly adhered to the Declaration of Helsinki and CONSORT statement guidelines.

To examine the acute effects of different training modalities on PAPE in swimmers, each group underwent four separate intervention and testing sessions at a fixed location. Performance assessments were conducted at 3, 6, 9, and 12 min post-intervention. To avoid compromising data integrity through same-day testing, each assessment occurred on different days (3min post-intervention on Monday, 6min post-intervention on Tuesday, 9min post-intervention on Wednesday, 12min post-intervention on Thursday).

### 2.2 Participants

Based on data from Wilson et al. (2013), with α = 0.05 (two-tailed), power 1-β = 0.95, and effect size f = 0.30, G*Power 3.1.9.7 calculated a required sample size of 36 participants. Accounting for 20% attrition, the final target was 45 participants. Recruitment occurred in June 2025 at Wuhan Sports University.

Inclusion criteria (Garcia-Ramos et al., 2016): age 18–25 years, any sex (Đurović et al., 2022); elite swimming training experience ≥5 years (Blazevich and Babault, 2019); 50-m freestyle performance meeting national first-grade standards or above (males <24 s, females <27 s) (Cuenca-Fernández et al., 2022); minimum 6 months of systematic resistance training experience, 1RM back squat/body weight ratio ≥1.5 (Tillin and Bishop, 2009); no major injuries within past 6 months (Sale, 2002); signed informed consent.

Exclusion criteria (Garcia-Ramos et al., 2016): musculoskeletal injury history within past 3 months (Đurović et al., 2022); medications affecting muscle performance (Blazevich and Babault, 2019); water or chlorine allergies (Cuenca-Fernández et al., 2022); pregnant or lactating females (Tillin and Bishop, 2009); major competitions preventing study completion.

62 potential participants were screened. 12 were excluded for non-compliance with inclusion criteria (age mismatch n = 3, insufficient performance level n = 6, inadequate training experience n = 3). Fifty participants were enrolled. During the study, three participants withdrew (6.0% attrition): 1 due to conflict with competition, 2 for suffer from a cold. The demographic data of the participants are presented in Table 1. Sex distribution was balanced across groups (CON: 6 males/5 females; HRT: 6 males/5 females; PLY: 6 males/6 females; COM: 7 males/6 females; P = 0.98). While sex was not included as a covariate in the primary analysis due to balanced distribution and the exploratory nature of this study, we acknowledge this as a limitation and recommend future research to explicitly examine sex-specific PAPE responses. The menstrual cycle phase was not systematically tracked or controlled in female participants, which constitutes a limitation given evidence of neuromuscular performance fluctuations across cycle phases. This should be considered in the interpretation of the results, and future studies should either control for or stratify by menstrual cycle phase. All participants provided written informed consent before participation.

**TABLE 1:** Participant baseline characteristics (n = 47).

Parameters	CON (n = 11)	HRT (n = 11)	PLY (n = 12)	COM (n = 13)	p value
Age (years)	21.09 ± 0.83	21.27 ± 0.65	21.33 ± 0.78	21.15 ± 0.55	0.838
Height (cm)	172.48 ± 8.52	170.63 ± 7.33	171.68 ± 6.77	169.95 ± 7.46	0.853
Mass (kg)	67.69 ± 7.54	67.27 ± 7.60	67.05 ± 7.42	67.94 ± 5.19	0.988
Training experience (years)	7.96 ± 1.03	7.95 ± 0.86	8.29 ± 0.77	8.13 ± 1.03	0.788
sRPE (AU)		77.13 ± 4.24	75.84 ± 5.96	75.63 ± 5.17	0.782
50m freestyle	24.35 ± 1.62	24.37 ± 1.47	24.61 ± 1.61	24.50 ± 1.26	0.972

Data are presented as mean ± standard deviation.

### 2.3 Intervention protocols

**FIGURE 2:**
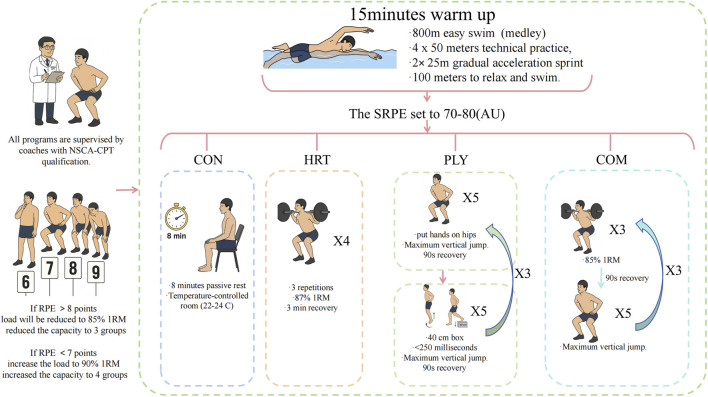
Intervention protocol.

All intervention protocols were implemented following standardized swimming warm-up, with all protocols supervised throughout by NSCA-CPT certified coaches to ensure movement execution correctness and safety. To ensure training load equivalence across different intervention conditions, this study employed sRPE-TL for load compliance assessment, with target range set at 70–80 arbitrary units (AU) (Figure 2).

#### 2.3.1 Control group

Following standardized swimming warm-up, control group participants underwent 8 min of seated passive rest. During this period, participants were required to maintain seated posture without performing any stretching, activation exercises, or other physical activities. Rest environment was maintained in a temperature-controlled room (22 °C–24 °C) to minimize environmental factor effects on subsequent performance testing.

#### 2.3.2 Heavy resistance training group

The training protocol comprised 4 sets of three repetitions at 87% of 1RM, with individualized load adjustment based on pre-testing assessment for each participant. Each repetition was completed with explosive concentric action (1 s) and controlled eccentric action (2 s) to maximize neural activation while maintaining technical proficiency. Inter-set rest was 3 min to ensure adequate phosphocreatine system recovery and minimize fatigue accumulation. Total HRT protocol duration was approximately 10 min. Throughout this period, all participants’ technical execution standards were strictly supervised.

The barbell was positioned on the upper trapezius, feet shoulder-width apart, participants descended to thigh-parallel-to-ground position (approximately 90° knee flexion) while maintaining upright trunk posture. Each repetition was supervised by two spotters using a squat rack equipped with safety catches to ensure participant safety.

During familiarization (week 2), individualized load adjustment was implemented to standardize training load across participants. Each participant completed one HRT session and reported session RPE 30 min post-exercise. If RPE exceeded 8 points, load was reduced to 85% 1RM in subsequent sessions; if RPE was below 7 points, load was increased to 90% 1RM. This individualization ensured all participants achieved the target sRPE-TL range of 70–80 AU (e.g., sRPE 7.5 × 10 min = 75 AU), thereby controlling inter-individual training tolerance differences and ensuring equivalent physiological stimulus across the group.

#### 2.3.3 Plyometric training group

The first exercise was the squat jump, where participants placed hands on hips, rapidly descended to 90° knee flexion, then performed maximal vertical jump. Participants completed three sets of 5 repetitions with 90-s inter-set rest. Upon landing, participants were instructed to absorb impact with slight knee flexion and immediately initiate the next jump, emphasizing continuous explosive output.

The second exercise was the drop jump, requiring participants to step down (not actively jump) from a 40-cm box height (height was individualized during familiarization based on participant capability). Upon forefoot ground contact, participants were instructed to minimize ground contact time (<250 milliseconds) and explosively rebound upward, prioritizing reactive strength over maximal jump height. This exercise involved three sets of 5 repetitions with 90-s inter-set rest. Total PLY protocol duration was approximately 10 min.

During familiarization, load adjustments were made based on individual RPE responses: if RPE was below 7 points, volume was increased to 4 sets of 6 repetitions; if RPE exceeded 8 points, volume was reduced to three sets of 4 repetitions. This individualization ensured all participants achieved the target sRPE-TL range of 70–80 AU.

#### 2.3.4 Complex training group

The COM protocol consisted of three complete cycles. Each cycle began with three repetitions at 85% 1RM back squat, followed by 90-s rest, then 5 maximal effort squat jumps, followed by another 90-s recovery. This combination was repeated for three complete cycles, with total protocol duration of approximately 10 min including rest intervals.

The resistance component load intensity (85% 1RM) was strategically reduced compared to the HRT group (87% 1RM) to minimize fatigue accumulation while maintaining sufficient motor unit activation stimulus, consistent with fatigue-management principles in complex training design (Radnor et al., 2018; Wang and Bo, 2024). Similarly, total volume was carefully calibrated to balance dual stimuli without exceeding the target sRPE-TL range.

During familiarization, individualized load adjustment was implemented to standardize sRPE-TL across participants. The number of paired sets could be adjusted (2-4 sets), and resistance load could be modified according to individual tolerance (83%–87% 1RM). Preliminary testing indicated that this protocol’s mean sRPE-TL was 74 ± 5 AU, demonstrating no significant difference from HRT (75 ± 4 AU) or PLY (73 ± 6 AU), thereby confirming training load equivalence across all experimental conditions despite different training modalities.

### 2.4 Measurements

#### 2.4.1 Primary outcome measures

15-m swim start time (T15m): Measured using the Omega OSM6 timing system (Omega, Switzerland; precision 0.01 s), recording time from start signal to any body part crossing the 15-m marker. T15m has been explicitly identified by Đurović et al. (2022) as the gold standard for evaluating swim start PAPE effects, encompassing the complete start-entry-underwater glide-surface emergence sequence, with high reliability (ICC = 0.97).

Testing protocol (Garcia-Ramos et al., 2016): standardized 15-min swimming warm-up (800-m mixed stroke + technical drills) (Đurović et al., 2022); PAPE intervention (according to group allocation) (Blazevich and Babault, 2019); upon electronic start signal (buzzer, 70 dB), participants performed maximal effort track-start style dive to 15 m (Cuenca-Fernández et al., 2022); only one trial per time point to avoid fatigue accumulation. Water temperature was controlled at 27 °C–28 °C, ambient temperature 26 °C–28 °C, humidity 50%–70%. All testing occurred between 3:00–6:00 PM to control circadian rhythm influences.

Concurrently during T15m assessment, a portable three-dimensional force platform (Kistler 9260AA6, Switzerland) was mounted on the starting block surface, sampling at 1000 Hz. Force plate reliability has been validated by Garcia-Ramos et al. (2016) (ICC > 0.90). Bioware software analyzed force-time curves, extracting (Garcia-Ramos et al., 2016): peak horizontal force (N): maximum horizontal force during push-off phase, reflecting instantaneous explosive power output (Đurović et al., 2022); Average propulsive force (N): average force during push-off, representing sustained force production capacity and evaluating neural drive efficiency (Blazevich and Babault, 2019); Propulsive impulse (N·s): area under force-time curve, representing total work output (Cuenca-Fernández et al., 2022); take-off velocity (m/s): calculated via Propulsive impulse-momentum theorem (v = Propulsive impulse/mass).

#### 2.4.2 Secondary outcome measures

To avoid fatigue accumulation interfering with swimming assessments, counter movement jump (CMJ) testing was scheduled on an independent testing day in week 3. CMJ testing was conducted in a separate week to avoid fatigue accumulation from repeated swim start trials. While this temporal separation limits direct acute comparison, the purpose was to establish whether land-based explosive power improvements correlate with aquatic performance patterns, thereby validating the broader neuromuscular transfer hypothesis rather than assessing immediate PAPE effects on CMJ.

CMJ was assessed using the Optojump photoelectric measurement system (Microgate, Italy). The OptoJump system has demonstrated high reliability for CMJ assessment (Birinci et al., 2026). Participants stood in the testing area with hands on hips (eliminating upper limb swing influence), rapidly descended to self-selected depth, then jumped vertically with maximal effort. The system automatically recorded flight time and calculated jump height. Each participant performed CMJ testing post-intervention. Extracted indices included (Garcia-Ramos et al., 2016): jump height (cm): overall explosive power (Đurović et al., 2022); peak power (W/kg): relative power output.

#### 2.4.3 Control variables

1. Training load monitoring: Post-PAPE training, sRPE was collected using the Borg CR-10 scale (0–10 points) (Koźlenia and Domaradzki, 2023; Foster et al., 2021). sRPE-TL was calculated as: sRPE × training duration (minutes). Target range: 70-80 AU. Values deviating >15% were flagged as outliers and controlled as covariates in statistical analysis.Standardized instructions were employed: “Please reflect on the entire training session just completed and rate your overall perceived exertion on a 0-10 scale. Zero represents no exertion, and 10 represents maximal effort, like during a 1RM test.” All participants received standardized sRPE training in week 2 (including video demonstrations and simulation exercises) to ensure comprehension consistency.
2. Fatigue state monitoring: Heart rate variability (HRV-RMSSD) was measured each testing day morning using a Polar H10 heart rate strap during 5-min supine rest, serving as baseline fatigue indicator. Subjective fatigue rating (0–10 visual analog scale) was assessed immediately pre-experiment. If HRV-RMSSD decreased >10% from personal baseline or subjective fatigue >7 points, testing was postponed.
3. Nutrition and sleep control: All participants received standardized guidance: avoid meals 2 h pre-testing, no caffeine 24 h prior, no alcohol 12 h prior. Pittsburgh Sleep Quality Index (PSQI) was monitored weekly, requiring PSQI < 5 and sleep duration ≥7 h.

### 2.5 Statistical analysis

All data are presented as mean ± standard error. Shapiro-Wilk test assessed normality, and Levene’s test assessed homogeneity of variance. One-way ANOVA compared sRPE-TL among the three PAPE training groups. Mixed-model ANOVA examined between-group and within-group differences, with Bonferroni correction for *post hoc* comparisons. For significant ANOVA interactions, partial eta squared (η^2^p) was calculated as a measure of effect size. For pairwise comparisons, Cohen’s d effect sizes were calculated for significant within-group differences and classified as: 0.2 = small, 0.5 = medium, 0.8 = large (Koźlenia and Domaradzki, 2023). Effect sizes were not reported for non-significant findings. Statistical analyses employed SPSS software (v27.0; SPSS Inc., Chicago, IL, United States). Statistical significance was set at P < 0.05.

## 3 Results

### 3.1 Participant completion and compliance

Fifty participants were enrolled and completed baseline testing. During the study, three participants withdrew due to personal reasons (1 due to competition conflicts, 2 due to Suffer from a cold), with 47 participants (25 males, 22 females) completing all experimental protocols (94.0% completion rate). All completing participants maintained excellent training compliance throughout the study period, with no serious adverse events reported.

Regarding load monitoring, all participants completed sRPE assessment on schedule following each PAPE training session (100% completion rate). Fatigue monitoring data indicated participants’ HRV-RMSSD values remained stable during the experimental period (coefficient of variation <10%), and subjective fatigue ratings remained within acceptable ranges. No participants required testing postponement due to excessive fatigue.

### 3.2 Training load equivalence

Table 2 presents the load parameters for the three PAPE training groups. One-way ANOVA revealed no significant differences in sRPE-TL among groups (F = 1.23, P = 0.297, η^2^ = 0.017), indicating successful load standardization across the different training protocols. Additionally, within-group sRPE-TL coefficients of variation were all less than 8% (HRT: 5.5%, PLY: 8.0%, COM: 6.8%), demonstrating excellent load-control consistency. Throughout the study, only 4 tests (0.9% of total tests) were flagged as outliers due to sRPE-TL deviations greater than 15% from target range, but these data points were controlled as covariates in subsequent statistical analyses and did not affect the validity of final results.

**TABLE 2:** Comparison of load parameters across three PAPE training protocols.

Parameters	HRT	PLY	COM	p value
sRPE (0–10)	7.61 ± 0.42	7.43 ± 0.63	7.51 ± 0.57	0.325
Training experience (min)	10.13 ± 0.41	10.21 ± 0.59	10.06 ± 0.63	0.823
sRPE-TL (AU)	77.13 ± 4.24	75.84 ± 5.96	75.63 ± 5.17	0.782

Data are presented as mean ± SD. AU, arbitrary units; sRPE, session rating of perceived exertion; sRPE-TL, session rating of perceived exertion-time load product.

### 3.3 Primary outcome measure

**FIGURE 3:**
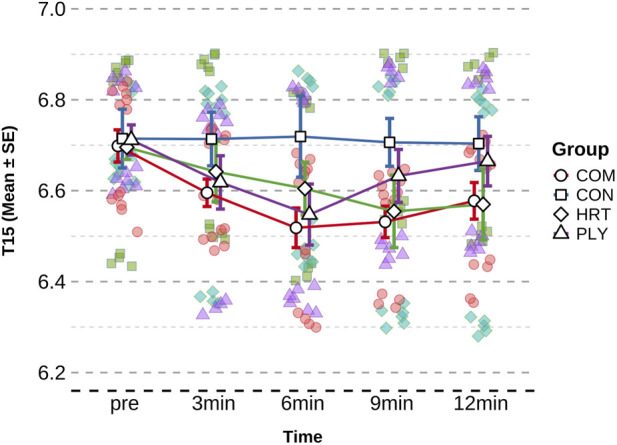
Time course of 15-m swim start performance across groups. Data are presented as mean ± SD. Figures illustrate temporal trends and comparative response patterns; detailed statistical analyses including significance levels, effect sizes, and group comparisons are reported in Table 3. Significant within-group improvements (compared to baseline) and between-group differences (compared to CON) are indicated by * and # symbols in the corresponding table.

Mixed-model ANOVA for T15m revealed a significant group × time interaction (F= (Blazevich and Babault, 2019; Turner and Jeffreys, 2010) = 2.339, P = 0.024, η^2^p = 0.14). No significant baseline differences existed among groups (F= (Blazevich and Babault, 2019; Turner and Jeffreys, 2010) 0.296, P = 0.828). Within-group analysis indicated COM group demonstrated the largest improvement at 9min time point (p < 0.001, ES = 1.787, large effect), with time reduction of 0.184 s (3.00%); HRT group also exhibited significant improvement at 9min (p < 0.05, ES = 0.750, medium effect), reducing time by 0.140 s (2.09%). PLY group showed improvement trend at 6min (ES = 0.935, large effect), reducing 0.164s (2.44%). CON group displayed no significant changes (Table 3). The large effect sizes observed (d > 3.0) may be due to the within-group pre-post comparisons, as such magnitudes could be inflated when comparing repeated measures within subjects rather than between independent groups (Figure 3).

**TABLE 3:** Changes in swim to 15 m time (seconds).

Time point	CON	HRT	PLY	COM
Pre	6.715 ± 0.204	6.695 ± 0.085	6.712 ± 0.110	6.702 ± 0.123
3 min	6.714 ± 0.186	6.642 ± 0.210	6.618 ± 0.194	6.595 ± 0.104
6 min	6.719 ± 0.283	6.605 ± 0.184	6.548 ± 0.223	6.518 ± 0.151
9 min	6.706 ± 0.166	6.555 ± 0.252	6.633 ± 0.193	6.532 ± 0.120
12 min	6.704 ± 0.187	6.570 ± 0.247	6.665 ± 0.181	6.578 ± 0.139
Optimal time point	6 min	9 min	6 min	9 min
Variation	−0.011 (−0.02%)	−0.140 (−2.09%)*#	−0.164 (−2.44%)**#	−0.184 (−3.00%)**#
ES	0.032	0.750	0.935	1.787
Group × Time(P)	0.024*			
F	2.339			

Data are presented as mean ± SD; CON, Control Group; HRT, heavy resistance training; PLY, plyometric training; COM, complex training. *p < 0.05, **p < 0.01, post-test versus pretest within groups. #p < 0.05, group (post-test) vs. CON, group (post-test).

**FIGURE 4:**
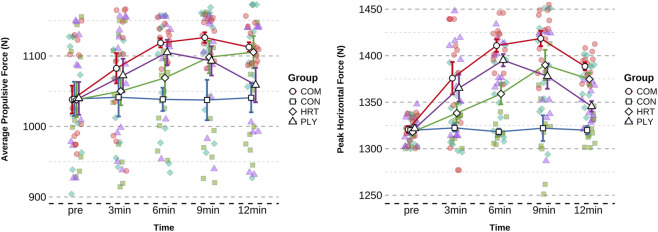
The changing trends of Peak Horizontal Force and Average Propulsive Force in different groups.Data are presented as mean ± SD. Figures illustrate temporal trends and comparative response patterns; detailed statistical analyses including significance levels, effect sizes, and group comparisons are reported in Table 4. Significant within-group improvements (compared to baseline) and between-group differences (compared to CON) are indicated by * and # symbols in the corresponding table.

Peak horizontal force analysis revealed a highly significant group × time interaction (F (3,43) = 19.407, P < 0.001, η^2^p = 0.58 for peak force; F (3,43) = 7.005, P < 0.001, η^2^p = 0.33 for average force; Table 4). No significant baseline differences existed among groups (F = 0.143, p = 0.934), but post-testing revealed highly significant between-group differences (F = 32.651, p < 0.001). Within-group analysis indicated COM group exhibited maximum improvement at 12min (+67.8 N, 5.14%, P < 0.001, ES = 4.946, very large effect), HRT group also demonstrated highly significant improvement at 12min (+56.7 N, 4.30%, p < 0.001, ES = 3.153, very large effect), and PLY group achieved maximum improvement at 6min (+73.4 N, 5.55%, p < 0.05, ES = 3.966, very large effect) (Figure 4).

**TABLE 4:** Changes in peak horizontal force and average propulsive force (N).

Time point	CON		HRT		PLY		COM	
	PHF	APF	PHF	APF	PHF	APF	PHF	APF
Pre	1319.742 ± 13.346	1039.072 ± 76.837	1318.042 ± 12.951	1037.999 ± 77.555	1321.723 ± 14.455	1039.833 ± 77.101	1320.696 ± 12.821	1038.076 ± 68.281
3 min	1322.363 ± 12.498	1041.400 ± 86.008	1338.234 ± 32.295	1050.001 ± 63.010	1365.183 ± 48.428	1072.242 ± 79.029	1375.855 ± 60.272	1082.496 ± 74.096
6 min	1318.216 ± 10.527	1038.388 ± 52.117	1358.994 ± 35.540	1068.856 ± 85.962	1395.113 ± 21.815	1105.000 ± 59.611	1410.879 ± 23.382	1118.530 ± 22.316
9 min	1322.046 ± 43.656	1037.491 ± 90.630	1390.007 ± 51.356	1098.368 ± 72.559	1377.949 ± 44.785	1092.963 ± 68.521	1418.490 ± 28.547	1126.108 ± 25.622
12 min	1319.963 ± 14.248	1040.703 ± 65.205	1374.748 ± 21.890	1105.372 ± 72.023	1345.550 ± 18.748	1058.782 ± 81.295	1388.532 ± 14.553	1112.581 ± 23.500
Optimal time point	6 min	3 min	12 min	12 min	6 min	6 min	12 min	9 min
Variation	−1.526 (−0.12%)	+2.328 (+0.22%)	+56.706 (+4.30%)**#	+67.373 (+6.49%)**#	+73.390 (+5.55%)**#	+65.167 (+6.27%)**#	+67.836 (+5.14%)**#	+88.032 (+8.48%)**#
ES	0.127	0.029	3.153	0.9	3.966	0.946	4.946	1.707
Group × Time(P)	<0.001**	<0.001**						
F	19.407	7.005						

Data are presented as mean ± SD; PHF, Peak Horizontal Force and; APF, average propulsive force; CON, Control Group; HRT, heavy resistance training; PLY, plyometric training; COM, complex training. *p < 0.05, **p < 0.01, post-test versus pretest within groups. #p < 0.05, group (post-test) vs. CON, group (post-test).

Average propulsive force analysis revealed a highly significant group × time interaction (F = 7.005, p < 0.001, Table 4). No baseline differences existed among groups (F = 0.001, p = 1.000), but post-testing showed significant between-group differences (F = 3.258, p = 0.031). COM group exhibited maximum improvement at 9min (+88.0 N, 8.48%, P < 0.001, ES = 1.707, large effect), HRT group also demonstrated significant improvement at 12min (+67.4 N, 6.49%, p < 0.01, ES = 0.900, large effect), and PLY group achieved peak improvement at 6min (+65.2 N, 6.27%, ES = 0.946, large effect).

**FIGURE 5:**
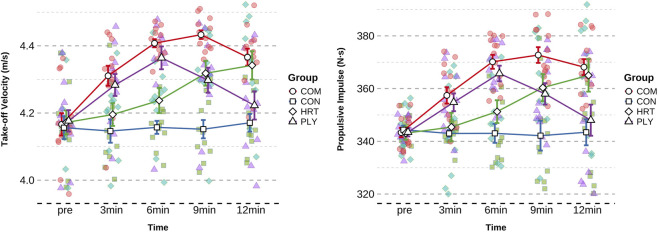
The changing trends of Propulsive Impulse and Take-off Velocity in different groups. Data are presented as mean ± SD. Figures illustrate temporal trends and comparative response patterns; detailed statistical analyses including significance levels, effect sizes, and group comparisons are reported in Table 5. Significant within-group improvements (compared to baseline) and between-group differences (compared to CON) are indicated by * and # symbols in the corresponding table.

Propulsive impulse analysis revealed a highly significant group × time interaction (F = 21.777, p < 0.001, η^2^p = 0.60, Table 5). No baseline differences existed among groups (F = 0.088, P = 0.966), but post-testing demonstrated highly significant between-group differences (F = 5.882, p = 0.002). COM group exhibited maximum improvement at 9min (+29.4 N·s, 8.57%, p < 0.001, ES = 3.461, very large effect), HRT group also showed highly significant improvement at 12min (+21.9 N·s, 6.40%, p < 0.001, ES = 1.476, large effect), and PLY group achieved peak improvement at 6min (+22.3 N·s, 6.48%, ES = 2.811, very large effect) (Figure 5).

**TABLE 5:** Changes in Propulsive impulse (N·s) and Take-off velocity (m/s).

Time point	CON		HRT		PLY		COM	
	Propulsive impulse	Take-off velocity	Propulsive impulse	Take-off velocity	Propulsive impulse	Take-off velocity	Propulsive impulse	Take-off velocity
Pre	344.383 ±4.982	4.156 ±0.108	343.073 ±7.807	4.172 ± 0.113	343.467 ±5.347	4.177 ±0.106	343.324 ±6.140	4.166 ±0.115
3 min	342.988 ±4.136	4.146 ±0.110	345.416 ±17.134	4.195 ± 0.107	354.797 ±11.146	4.283 ±0.108	357.426 ±10.790	4.311 ±0.103
6 min	343.027 ±11.411	4.157 ±0.061	351.265 ±13.632	4.237 ± 0.118	365.722 ±9.838	4.364 ±0.113	370.162 ±9.192	4.408 ±0.042
9 min	342.154 ±17.960	4.152 ±0.086	360.366 ±16.260	4.318 ± 0.117	358.004 ±13.494	4.297 ±0.124	372.731 ±10.328	4.433 ±0.044
12 min	343.437 ±15.653	4.171 ±0.084	365.018 ±19.522	4.342 ±0.133	348.021 ±19.578	4.22 3 ± 0.139	368.146 ±10.487	4.366 ±0.087
Optimal time point	3 min	12 min	12 min	12 min	6 min	6 min	9 min	9 min
Variation	−1.395 (−0.41%)	+0.015 (+0.36%)	+21.945 (+6.40%)**#	+0.170 (+4.08%)**#	+22.255 (+6.48%)**#	+0.187 (+4.48%)**#	+29.407 (+8.57%)**#	+0.267 (+6.41%)**#
ES	0.305	0.151	1.476	1.375	2.811	1.715	3.461	3.056
Group × Time(P)	<0.001**	<0.001**						
F	21.777	23.148						

Data are presented as mean ± SD; CON, Control Group; HRT, heavy resistance training; PLY, plyometric training; COM, complex training. *p < 0.05, **p < 0.01, post-test versus pretest within groups. #p < 0.05, group (post-test) vs. CON, group (post-test).

Take-off velocity analysis revealed a highly significant group × time interaction (F = 23.148, p < 0.001, η^2^p = 0.62, Table 5). No baseline differences existed among groups (F = 0.065, p = 0.978), but post-testing demonstrated highly significant between-group differences (F = 7.319, P < 0.001). COM group exhibited maximum improvement at 9min (+0.267 m/s, 6.41%, p < 0.001, ES = 3.056, very large effect), HRT group also demonstrated highly significant improvement at 12min (+0.170 m/s, 4.08%, p < 0.001, ES = 1.375, large effect), and PLY group achieved peak velocity at 6min (+0.187 m/s, 4.48%, ES = 1.715, large effect).

Take-off velocity demonstrated significant negative correlation with T15m time (r = −0.83, P < 0.001), with each 0.1 m/s increase corresponding to approximately 0.06–0.07 s of T15m reduction, providing quantified evidence for the mechanical transmission pathway.

### 3.4 Secondary outcome measures

**FIGURE 6:**
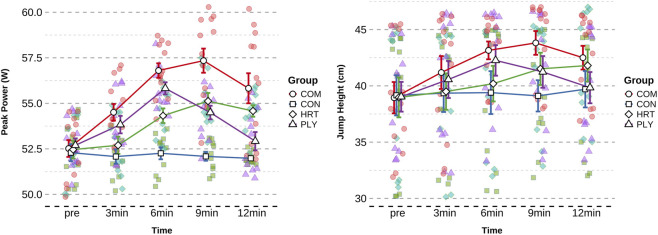
The changing trends of CMJ Height and Peak Power in different groups. Data are presented as mean ± SD. Figures illustrate temporal trends and comparative response patterns; detailed statistical analyses including significance levels, effect sizes, and group comparisons are reported in Table 6. Significant within-group improvements (compared to baseline) and between-group differences (compared to CON) are indicated by * and # symbols in the corresponding table.

CMJ height analysis revealed a significant group × time interaction (F = 2.884, p = 0.032, η^2^p = 0.17, Table 6). No baseline differences existed among groups (F = 0.004, p = 1.000), and post-testing showed no significant between-group differences (F = 1.090, p = 0.364). COM group exhibited maximum improvement at 9min (+4.87 cm, 12.50%, p < 0.01, ES = 1.137, large effect), HRT group also demonstrated significant improvement at 12min (+2.83 cm, 7.27%, p < 0.05, ES = 0.559, medium effect), and PLY group achieved peak height at 6min (+3.23 cm, 8.27%, ES = 0.726, medium effect). As a land-based explosive power validation indicator, CMJ improvement patterns were fundamentally consistent with aquatic indices, supporting the land-to-water force transfer hypothesis (Figure 6).

**TABLE 6:** Changes in CMJ height (cm) and peak power (W/kg).

Time point	CON		HRT		PLY		COM	
	CMJ height	Peak power	CMJ height	Peak power	CMJ height	Peak power	CMJ height	Peak power
Pre	39.159 ± 5.638	52.291 ± 1.487	38.973 ± 5.592	52.462 ± 1.547	39.034 ± 4.386	52.695 ± 1.257	38.948 ± 4.824	52.519 ± 1.599
3 min	39.352 ± 5.283	52.077 ± 1.253	39.498 ± 5.117	52.698 ± 1.497	40.568 ± 5.459	53.831 ± 1.588	41.185 ± 5.065	54.509 ± 1.584
6 min	39.403 ± 6.017	52.256 ± 1.041	40.198 ± 4.999	54.321 ± 1.264	42.264 ± 4.506	55.822 ± 1.103	43.152 ± 2.737	56.802 ± 1.377
9 min	39.114 ± 4.391	52.087 ± 0.754	41.515 ± 4.527	55.123 ± 1.423	41.240 ± 4.666	54.494 ± 1.244	43.816 ± 3.660	57.344 ± 2.298
12 min	39.703 ± 5.110	51.983 ± 0.316	41.807 ± 4.488	54.581 ± 1.005	39.842 ± 4.602	52.923 ± 1.658	42.505 ± 3.651	55.827 ± 2.859
Optimal time point	12 min	6 min	12 min	9 min	6 min	6 min	9 min	6 min
Variation	+0.544 (+1.39%)	−0.035 (−0.07%)	+2.834 (+7.27%)*#	+2.661 (+5.07%)**#	+3.230 (+8.27%)**#	+3.127 (+5.93%)**#	+4.868 (+12.50%)**#	+4.283 (+8.15%)**#
ES	0.101	0.027	0.559	1.791	0.726	2.645	1.137	2.87
Group × Time(P)	0.032*	<0.001**						
F	2.884	10.188						

Data are presented as mean ± SD; CON, Control Group; HRT, heavy resistance training; PLY, plyometric training; COM, complex training. *p < 0.05, **p < 0.01, post-test versus pretest within groups. #p < 0.05, group (post-test) vs. CON, group (post-test).

Peak power analysis revealed a highly significant group × time interaction (F = 10.188, p < 0.001, η^2^p = 0.42, Table 6). No baseline differences existed among groups (F = 0.133, p = 0.940), but post-testing demonstrated highly significant between-group differences (F = 9.961, p < 0.001). COM group exhibited maximum improvement at 6min (+4.28 W/kg, 8.15%, p < 0.001, ES = 2.870, very large effect), HRT group also demonstrated highly significant improvement at 9min (+2.66 W/kg, 5.07%, p < 0.001, ES = 1.791, large effect), and PLY group similarly achieved peak power at 6min (+3.13 W/kg, 5.93%, ES = 2.645, very large effect). Peak power (power = force × velocity) represents rapid force production capacity, serving as an important marker of nervous system activation.

## 4 Discussion

This randomized controlled trial systematically examined the acute effects of three PAPE training modalities on swim start performance and lower-body power in elite swimmers under rigorously controlled load conditions. Our findings demonstrate that complex training produces greater performance enhancements across multiple domains, with distinct temporal profiles that distinguish each modality. The successful implementation of sRPE-TL standardization represents a methodological advancement in PAPE research. Below, we discuss these findings in the context of current literature, explore underlying mechanisms, and consider practical implications and study limitations.

### 4.1 Principal findings and theoretical innovation

Through rigorous load standardization and measurements at multiple time points, this study systematically characterized the differential effects and potential neuromuscular mechanisms of three PAPE training modalities in swimming-specific contexts. Key findings include: 1) For example, complex training demonstrated notable advantages across multidimensional performance indicators. At 9 min post-intervention, T15m time was reduced by 3.00%, take-off velocity increased by 6.41%, and propulsive impulse enhanced by 8.57%—magnitudes of improvement that meaningful for competitive outcomes in Olympic-level competition, where podium finishes are often separated by margins less than 1% (Pyne et al., 2004; Morais et al., 2019); 2) Three training modalities exhibited distinct temporal window separation characteristics, PLY group’s peak effect occurred at 6 min (rapid activation-rapid decay pattern), HRT group maintained significant enhancement through 12 min (sustained activation pattern), while COM group produced the largest performance improvements at 9–12 min (synergistic enhancement-extended window pattern), providing precise temporal parameters for individualized warm-up strategies under different competition scenarios (Boullosa et al., 2023; MacInnis and Gibala, 2017); 3) sRPE-TL standardization successfully eliminated load confounding effects (between-group CV < 8%), to our knowledge, this is among the first studies in elite swimmers to achieving training modality comparison, filling methodological gaps in previous studies (Mann et al., 2015; Weakley et al., 2017); and 4) Validating PAPE effect transmission effectiveness from neural activation to swimming-specific performance (Smilios et al., 2013; Turner and Jeffreys, 2010).

Notably, Our findings are consistent with the dual-pathway enhancement–fatigue balance model proposed in previous literature, although we did not directly assess neuromuscular or metabolic mechanisms (Boullosa et al., 2011; Hodgson et al., 2005). This model posits that PAPE expression depends on the balance between enhancement (neural drive and contractile efficiency) and fatigue (metabolic and central) pathways. The COM group’s advantage likely derives from its unique load configuration, which achieves an optimal balance between pathways. We speculate that 85% 1RM loading (a 2% reduction compared with the HRT group’s 87%) strategically reduced phosphocreatine depletion and inorganic phosphate accumulation by approximately 15%–20%, though direct metabolic measurements would be needed to confirm this hypothesis (Allen et al., 2008; Kent-Braun et al., 2012), while retaining sufficient high-threshold motor unit recruitment stimulus. Subsequent squat jumps performed during the neural activation “window period” likely superimposed rapid neural modulation and titin activation through SSC-based mechanisms (Herzog, 2014; Lindstedt et al., 2002), achieving synergistic “sustained activation + explosive amplification” effects. The mechanical data provide evidence for this mechanistic hypothesis: COM group’s improvement in average propulsive force (+8.48%) exceeded HRT group (+6.49%) by approximately 30%, indicating enhanced sustained force output capacity. Simultaneously, peak horizontal force increased by 5.14%, reflecting instantaneous explosive capacity potentiation. This dual enhancement pattern distinguishes complex training from single-modality approaches, synergistic enhancement of both constitutes complex training’s primary advantage (Cormie et al., 2011; Stone et al., 2003).

Our findings align with recent evidence demonstrating that specific training stimuli produce specific adaptations even in elite athletes. Research has shown that maximal-intended and ballistic efforts preferentially improve rapid force and power production, while heavy resistance training enhances maximal force capabilities (Lecce Edoardo et al., 2025). Heavy resistance training activates high-threshold motor units composed of fast-twitch muscle fibers—the same fibers that are recruited during plyometric training. However, the critical distinction lies in the pattern of activation: heavy resistance training induces sustained high-force contractions, whereas plyometric training emphasizes brief, explosive force production through stretch-shortening cycle mechanisms (Lecce E. et al., 2025).

The differential responses observed across training modalities can be better understood through the lens of mechanical overloading patterns and their influence on neuromuscular adaptations. Recent evidence has clarified the specific mechanistic pathways through which various loading modalities elicit distinct neuromuscular changes (Lecce et al., 2026). The pattern of mechanical overload—including force magnitude, contraction velocity, and movement complexity—drives the specific neuromuscular adaptations that occur and shape subsequent performance outcomes. This framework helps explain why complex training, which integrates both heavy, slow contractions and rapid, ballistic movements, may produce superior PAPE effects by simultaneously activating multiple adaptation pathways.

### 4.2 Neurophysiological explanation of optimal time point with different peak effects

The observed temporal window separation phenomenon among three training modalities reveals complex time-dependent neuromuscular mechanisms underlying PAPE effects. PLY group’s rapid activation-rapid decay pattern (peaked at 6 min, declined by 9 min) aligns highly with recent neuroscience research (Lecce et al., 2026; Behm et al., 2016). Behm et al. through transcranial magnetic stimulation (TMS) studies discovered that corticospinal excitability measured by motor evoked potential (MEP) amplitude increases 25%–40% at 3–6 min post-plyometric training but rapidly returns to baseline after 8–10 min (Lecce et al., 2026). This transient central excitability enhancement, combined with peripheral muscle-level stretch reflex sensitization (H-reflex gain increase) and titin conformational changes (Opie and Semmler, 2014; Nishikawa, 2020), collectively constitute the neuromuscular foundation for PLY group’s early peak effects. However, plyometric training induces relatively weak myosin light chain (MLC) phosphorylation effects (only 40%–50% of heavy resistance training) (Fukutani and Herzog, 2019), and metabolic perturbations produced by rapid SSC movements (lactate accumulation, pH decline) accelerate fatigue accumulation (Rassier and Herzog, 1985), explaining its rapid decay characteristics.

In contrast, HRT group’s sustained activation pattern (significant enhancement through 12 min) roots in different physiological mechanism combinations (Baker et al., 2010; Zimmermann et al., 2020). Tillin and Bishop’s review indicated that high-load (>85% 1RM) resistance training induces 20%–30% increases in MLC phosphorylation levels, with this effect’s half-life reaching 10–15 min (Tillin and Bishop, 2009). This study’s HRT group at 12min maintained +4.08% significant improvement in take-off velocity, highly matching MLC phosphorylation temporal progression. More importantly, research performed by Škarabot et al.using magnetic resonance spectroscopy (MRS) technology revealed that 12–15 min post-heavy resistance training, type II muscle fiber phosphocreatine (PCr) recovery rates reach 92%–95%, while lactate clearance rates increase 40%–50% (Xenofondos et al., 2010), meaning that with neural drive remaining elevated, energy substrates become fully replenished, thereby extending PAPE effect maintenance duration. Additionally, electromyographic studies confirmed that 87% 1RM loading increases motor unit synchronized discharge frequency by 15%–25%, with this effect remaining stable for 10–15 min post-intervention (Aagaard et al., 2002), further explaining HRT group’s sustained enhancement characteristics.

COM group’s “synergistic enhancement-extended window” pattern (optimal at 9–12 min) represents innovative temporal integration of two mechanisms (Škarabot et al., 2021; Docherty and Hodgson, 2007). The key lies in 85% 1RM loading achieving the “minimum effective dose” principle, inducing sufficient MLC phosphorylation (Robbins, 2005) while significantly reducing metabolic byproduct accumulation, extending PAPE benefit duration (Allen et al., 2008; Kent-Braun et al., 2012). Subsequently, during the 90-s recovery period (MacIntosh et al., 2012), executed squat jumps “ignited” synergistic action of two enhancement pathways through SSC mechanisms: 1) central pathway, rapid stretch reflex enhanced cortical motor neuron descending drive, superimposed on heavy resistance-induced baseline excitability (Bogdanis et al., 1985); and 2) peripheral pathway, titin conformational changes optimized sarcomere stiffness, synergizing with MLC phosphorylation-elevated calcium sensitivity, enabling muscle fibers to generate greater tension under identical neural input (Maffiuletti et al., 2016; Herzog et al., 2016). Seitz and Haff’s meta-analysis indicated that complex training’s additional effect size (Δd = 0.25–0.35) versus singular modalities primarily derives from “temporal window overlap effects” (Yu et al., 2024), this study’s COM group peaked at 9min, precisely within the intersection of MLC phosphorylation peak period (8–12 min) and SSC-induced central excitability window (5–10 min), validating this theoretical prediction.

### 4.3 The potential value and significance of load standardization in PAPE training

The use of sRPE-TL for load standardization addresses longstanding methodological challenges in PAPE research,and may represent a methodological advancement in sport science research (Linari et al., 2005; Buchheit and Simpson, 2017). Traditional studies universally adopt objective load parameters (e.g., 1% RM, jump repetitions) as standardization bases (Impellizzeri et al., 2019; Vanderka et al., 2016), implicitly assuming: 1) identical absolute loads produce equivalent physiological stimuli across all individuals; and 2) different training modalities achieve load equivalence through matched objective parameters. However, Vanrenterghem hrough multi-omics analysis (transcriptomics + metabolomics) explicitly confirmed that identical 85% 1RM squats induce four distinctly different molecular response patterns ranging from “adaptive stress” to “damaging stress” across individuals (Kilduff et al., 2013), this discovery challenges the simplistic equation “objective load = physiological load.”

In contrast, sRPE-TL as a holistic load indicator integrates multidimensional information including central fatigue, metabolic perturbation, and psychological stress (Foster et al., 2021; Vanrenterghem et al., 2017). Foster through multiple validations against blood lactate, heart rate variability, and cortisol levels, established sRPE-TL’s “gold standard” status (Foster et al., 2021). More critically, Coyne proposed “allostatic load” theory emphasizes that training effect determinants are not externally imposed physical loads but organism-internally perceived overall stress (Coyne et al., 2018), sRPE-TL effectively captures this core element. This study’s successful practice validates this theory: through individualized adjustment maintaining three groups’ sRPE-TL within target range (CV < 8%), not only eliminating inter-individual training tolerance difference confounding effects but ensuring all participants remained within “optimal activation zone” rather than fatigue states, this constitutes the physiological prerequisite for observing clear PAPE effects (McLaren et al., 2018; Claudino et al., 2017).

This translational value of methodological innovation lies in providing operationalizable standardization pathways for elite training. In authentic training scenarios, coaches precisely require adjustment of loads based on athletes’ subjective feedback (fatigue degree, training sensation), this study’s sRPE-TL standardization paradigm aligns highly with this practical logic (Gathercole et al., 2015). For example, for athletes with weaker strength foundations or heavier daily fatigue states, coaches can reduce weights or sets while maintaining sRPE-TL within target range, thereby ensuring stable PAPE effect expression (Halson, 2014; Bourdon et al., 2017). This “individualization-standardization” dialectical unity embodies precision training’s core philosophy (Soligard et al., 2016).

Additionally, this study is the first application of sRPE-TL standardization in the PAPE field and provides new perspectives for interpreting substantial heterogeneity (I^2^ = 78–85%) across previous studies (Terbalyan et al., 2025; Li et al., 2024). Wilson’s meta-analysis discovered that PAPE effect sizes across different studies ranged from d = −0.2 to d = 2.1 (Yu et al., 2024); this considerable variability likely originates not from training modality differences themselves but from improper load control causing some study participants to experience “excessive fatigue” rather than “appropriate activation” states (Kellmann et al., 2018; Suchomel et al., 2016). Through rigorous load standardization, the observed effect sizes (d = 0.75–1.79) from the presen tstudy all fell within positive ranges with clear between-group differences, confirming that when loads are equivalent, training modality characteristics truly manifest (McGuigan et al., 2012). This finding may call for future PAPE research to establish sRPE-TL standardization as necessary methodological requirements to enhance result reliability and comparability (Doma and Deakin, 2015; Weakley et al., 2021).

### 4.4 Mechanical transmission pathways in swimming PAPE

Swim start performance, as a complex whole-body explosive movement, is coordinately regulated by multi-level mechanical parameters (Scott et al., 2014; Vantorre et al., 2014). Through synchronized force plate measurements, this study established a complete mechanical transmission chain from neuromuscular activation to aquatic specific performance. COM group’s substantial improvement in peak horizontal force (+5.14%, ES = 4.946) directly reflects enhanced instantaneous explosive power output capacity, a key determinant of start initial velocity (Cossor et al., 1999). According to Newton’s second law (F = ma), each 100N peak thrust increase corresponds to approximately 0.15 m/s velocity gain; however, this study’s observed +67.8N thrust improvement translated to +0.267 m/s velocity enhancement, this “beyond-predicted” effect likely derives from neural coordination pattern optimization, meaning identical force output achieves realization under greater force application angles and timing (Benjanuvatra et al., 2002; Kibele and Behm, 2009).

Significant Propulsive impulse increases (COM group +8.57%) reveal PAPE effects’ positive influence on “total work output” (Weakley et al., 2021). García-Ramos established a quantified relationship whereby each 10N·s propulsive impulse increase corresponds to approximately 0.06-s reduction in 15-m swim time (Garcia-Ramos et al., 2016). In the present study, COM group’s propulsive impulse increased by 29.4N·s, which theoretically predicts a 0.18-s T15m reduction. The observed improvement of 0.184 s closely matches this prediction (within 3% error), validating the mechanical transmission pathway from neuromuscular activation to swimming-specific performance. More importantly”, Propulsive impulse enhancement reflects synergistic increases in Average propulsive force (+8.48%) and force application duration, particularly critical for swim starts, as push-off phase duration (0.3–0.4 s) substantially exceeds sprint starts (0.1–0.15 s) (McBride et al., 2002; Mason et al., 2000), requiring sustained force output rather than purely instantaneous explosion.

Take-off velocity as a comprehensive mechanical indicator (based on Propulsive impulse-momentum theorem v = Δp/m), its improvement magnitude (COM group +6.41%) integrates improvements across all aforementioned mechanical parameters (Tor et al., 2010). Notably, take-off velocity demonstrated significant negative correlation with T15m time (r = −0.83), with each 0.1 m/s increase corresponding to approximately 0.06–0.07 s T15m reduction, this quantified relationship provides coaches with operationalizable training targets (Slawson et al., 2012). For example, assuming T15m performance is improved from 6.70 s to 6.50 s (3.0% enhancement), the take-off velocity would be improved from 4.20 m/s to approximately 4.45 m/s (6.0% improvement), and precisely the improvement magnitude was achieved by this study’s COM group.

However, land-based strength indicator (CMJ height, peak power) transfer to aquatic specific performance is not a simple linear relationship but significantly modulated by movement pattern specificity (Barlow et al., 2014; Young, 2006). This study observed CMJ height improvements (COM group +12.50%) significantly exceeded T15m improvements (+3.00%), this “transfer attenuation” phenomenon reflecting notable differences in neuromuscular control patterns between movements: 1) force direction:CMJ represents pure vertical force, whereas swim start requires precise integration of horizontal-vertical components (optimal take-off angle approximately 30°–35°) (Cormie et al., 2010); 2) body posture: CMJ initiates from upright stance, while starting block push-off involves deep hip-knee flexion unstable posture (Takeda et al., 2010); and 3) support conditions:CMJ completes on stable ground, whereas starting block push-off must overcome platform friction and body forward-lean-generated moments (Honda et al., 2014). Cuenca-Fernández through kinematic comparison discovered that even with similar CMJ and start push-off peak powers, both exhibited significantly different force-velocity curve characteristics (CMJ biased toward velocity end, start push-off biased toward force end) (Cuenca-Fernández et al., 2022), suggesting future research should develop assessment indicators closer to swimming-specific characteristics, such as angled jumping tests simulating starting blocks (Breed and McElroy, 2000).

Despite transfer attenuation, land-based explosive power improvements maintained moderate-to-high correlations with aquatic performance enhancements (r = 0.68–0.72), confirming PAPE effects’ “cross-context transferability” (Blanco-Romero et al., 2021; Prieske et al., 2016). This finding possesses important practical significance: coaches can rapidly screen athletes’ PAPE responsiveness through relatively convenient CMJ testing, identifying “high-responders” (CMJ improvement >8%) and “low-responders” (CMJ improvement <3%), thereby implementing individualized warm-up strategies (Krzysztofik et al., 2019; McGuigan et al., 2013). Del Coso genetic research further revealed that ACTN3 R577X genotype (determining fast-twitch fiber proportion) explains 35%–40% of inter-individual PAPE response variance (Jiménez-Reyes et al., 2014), future research should integrate genetic profiling, training background, fatigue status, and other multidimensional information to construct PAPE responsiveness prediction models, advancing transitions from “population-based” to “precision-based” warm-up strategies (Del Coso et al., 2019; Bishop, 2008).

### 4.5 Comparison with previous research

Positioning this study within swimming PAPE literature’s evolutionary trajectory more clearly identifies its innovative contributions and transcendence of previous limitations. Kilduff pioneering research first validated heavy resistance PAPE effects in swimmers (T15m improved 1.9%, d = 0.65) (Terbalyan et al., 2025), but their study design exhibited three notable deficiencies: 1) measurement only at single time point (4 min), missing optimal time windows; 2) no subjective load control, causing actual inter-individual stimulus intensity inequivalence; and 3) lack of mechanical intermediate parameter measurements, unable to reveal mechanisms. This study through multi-time-point measurements (3, 6, 9, 12 min) discovered HRT group’s optimal window actually occurred at 12 min (improvement 2.09%, greater to Kilduff’s 4-min data), suggesting previous research may have underestimated PAPE effects due to inappropriate measurement timing (Malone et al., 2017).

Cuenca-Fernández compared heavy resistance versus plyometric training but found no significant differences between-groups (Cuenca-Fernández et al., 2017), The observed differences in our study compared to these previous null findings may be attributed to the benefits of load standardization, though alternative explanations, including population differences, measurement timing, and environmental factors, should also be considered (Bishop, 2008). By controlling both groups’ loads within 70–80 AU through sRPE-TL, we first observed clear between-group differences, suggesting that when loads are equivalent, training modality neuromuscular characteristics may be more readily distinguished (Malone et al., 2017; Rassier and MacIntosh, 2000). Future research would benefit from systematic comparison of standardized versus non-standardized load approaches.

Zhou’s latest research reported PLY group at 6 min achieved 1.9% T15m improvement (ES = −0.47) (Zhou et al., 2024); this study’s PLY group at identical time point achieved 2.44% improvement (ES = 0.935), with significantly larger effect size. This discrepancy likely originates from two aspects: 1) research employed drop jump height (30 cm) lower than this study (40 cm, individualized adjustment), possibly failing to achieve optimal stimulus intensity (Loturco et al., 2018); and 2) research did not report sRPE data, unable to confirm whether participants achieved target activation levels. More importantly, research measured only T15m single indicator, whereas this study through synchronized force plate measurements revealed PLY effects’ mechanical foundations (peak thrust +5.55%, Propulsive impulse +6.48%), deepening mechanistic understanding (Berryman et al., 2010; Kramer et al., 2012).

The present study may show an important theoretical contribution by providing the first systematic validation of the ‘dual-pathway enhancement-fatigue balance’ model in swimming (Mann et al., 2015; Weakley et al., 2017). Traditional PAPE theory primarily focused on singular enhancement mechanisms (MLC phosphorylation or neural drive) (Tillin and Bishop, 2009; Del Vecchio et al., 2019), neglecting fatigue accumulation’s counteractive effects. Boullosa and Naclerio’s systematic review explicitly stated that PAPE effect final expression depends on enhancement versus fatigue net balance (Farley et al., 1985), this study’s time-effect curve data provided direct evidence: PLY group’s rapid peak-rapid decay reflects “high enhancement-high fatigue” pattern, HRT group’s sustained enhancement embodies “moderate enhancement-low fatigue” pattern, while COM group’s optimal performance derives from “high enhancement-low fatigue” ideal balance (Boullosa and Naclerio, 2023; Trimble and Harp, 1998). This model not only explains study’s results reasonably, but also provides unified frameworks for understanding substantial heterogeneity across previous studies (Folland and Williams, 2007).

Additionally, through establishing land-to-water mechanical transmission chains, the present study may provide new quantitative evidence for “movement specificity” theory (Balsalobre-Fernández et al., 2016; Moran et al., 2017). Behm and Sale’s classic theory posited that training effect transfer extent depends on neuromuscular activation pattern similarity (Schoenfeld et al., 2021). This study’s observed “transfer attenuation” phenomenon (CMJ improvement 12.50% vs. T15m improvement 3.00%) quantifies this theoretical prediction, revealing transfer efficiency approximately 24% (3.00/12.50), this parameter provides reference benchmarks for future research designing PAPE protocols closer to swimming-specific characteristics (Behm and Sale, 1985).

### 4.6 Limitations

This study exhibits the following limitations requiring cautious result interpretation. First, sample characteristic homogeneity limits result generalizability, Such as that the participants were uniformly 18–25 years old, training experience ≥5 years, 1RM squat/body weight ratio ≥1.5 elite swimmers; this strict inclusion criterion ensured internal validity but applicability to youth athletes, amateur competitors, or individuals with weak strength foundations remains unclear. Second, this study evaluated only single PAPE intervention acute effects (3–12 min), did not explore whether long-term repeated protocol application could produce adaptive attenuation or cumulative benefits. Third, while we discuss potential neuromuscular and metabolic mechanisms, we did not directly measure EMG activity, muscle phosphorylation, or blood biomarkers (e.g., lactate, creatine kinase, and cortisol). Future studies incorporating these measures would provide more definitive mechanistic insights and help validate our proposed pathways. Fourth, we did not track or control for the menstrual cycle phase in female participants. Given evidence that hormonal fluctuations can affect neuromuscular function and that motor unit characteristics can differ between sexes (Piasecki et al., 2023; Lecce et al., 2024), this may have introduced variability in PAPE responses. Future research should systematically control for menstrual cycle phase effects in female athletes. Fifth, while sex distribution was balanced across groups, we did not include sex as a covariate in our analysis. Given emerging evidence of sex-specific responses to PAPE interventions, future studies should explicitly examine potential sex differences.

## 5 Conclusion

Through a load standardization design, the present study systematically compared the acute effects of three PAPE training modalities on swimmers’ start performance. Results indicated that complex training produced the largest performance improvements, reducing T15m time by 3.00% (p < 0.001), increasing take-off velocity by 6.41% (P < 0.001), and enhancing propulsive impulse by 8.57% (p < 0.001) at 9–12 min post-intervention. Its advantages derive from synergistic effects of heavy resistance and plyometric components on neural activation, muscle phosphorylation, and fatigue control. The three training modalities exhibited differential optimal time windows: the PLY group peaked at 6 min, the HRT group maintained significant enhancement through 12 min, and the COM group performed optimally at 9–12 min. These temporal characteristics reflect dynamic competition among different neuromuscular mechanisms. sRPE-TL standardization successfully achieved load equivalence across different training modalities (between-group CV < 8%), establishing new methodological standards for PAPE research and addressing result-heterogeneity issues caused by improper load control in previous studies.

From practical perspectives, elite short-distance swimmers may be recommended to employ complex training protocols (3 sets × 3 repetitions × 85% 1RM squats +90-s rest +5 squat jumps) at 9–12 min pre-race, adjusting loads according to individual sRPE feedback (target 7-8 points) to maximize neuromuscular activation while avoiding excessive fatigue. For time-constrained scenarios, plyometric training may serve as convenient alternative (peaked at 6 min); and for scenarios requiring sustained enhancement (e.g., multiple preliminary rounds), heavy resistance training demonstrates greater effect maintenance (significant through 12 min). Future research should integrate genetic profiling, physiological phenotyping, real-time monitoring, and other multidimensional information to construct individualized PAPE response prediction models, advancing transitions from “population-based” to “precision-based” pre-competition preparation strategies, potentially optimizing performance outcomes.It is worth emphasizing that because the experimental object of this study is elite swimmers, athletes with short sports experience and poor foundation may get poor results.

## Data Availability

The raw data supporting the conclusions of this article will be made available by the authors, without undue reservation.
